# The Destructive Cycle in Bronchopulmonary Dysplasia: The Rationale for Systems Pharmacology Therapeutics

**DOI:** 10.3390/antiox14070844

**Published:** 2025-07-10

**Authors:** Mia Teng, Tzong-Jin Wu, Kirkwood A. Pritchard, Billy W. Day, Stephen Naylor, Ru-Jeng Teng

**Affiliations:** 1 Department of Integrative Biology, University of Wisconsin-Madison, 145 Noland Hall, 250 N. Mills St., Madison, WI 53706, USA; mteng7@wisc.edu; 2 Department of Pediatrics, Medical College of Wisconsin, Suite C410, Children’s Corporate Center, 999 N. 92nd Street, Milwaukee, WI 53226, USA; twu@mcw.edu; 3 Department of Surgery, Medical College of Wisconsin, 8701 Watertown Plank Rd., Milwaukee, WI 53226, USA; kpritch@mcw.edu; 4 ReNeuroGen LLC, 2160 San Fernando Dr, Elm Grove, WI 53122, USA; billy.day@rngen.com (B.W.D.); snaylor@rngen.com (S.N.)

**Keywords:** bronchopulmonary dysplasia, systems pharmacology therapeutic, unfolded protein response, mitochondria, inflammation, cellular senescence, endothelial nitric oxide synthase

## Abstract

Bronchopulmonary dysplasia (BPD) remains a significant complication of premature birth and neonatal intensive care. While much is known about the drivers of lung injury, few studies have addressed the interrelationships between oxidative stress, inflammation, and downstream events, such as endoplasmic reticulum (ER) stress. In this review, we explore the concept of a “destructive cycle” in which these drivers self-amplify to push the lung into a state of maladaptive repair. Animal models, primarily the hyperoxic rat pup model, support a sequential progression from the generation of reactive oxygen species (ROS) and inflammation to endoplasmic reticulum (ER) stress and mitochondrial injury. We highlight how these intersecting pathways offer not just therapeutic targets but also opportunities for interventions that reprogram system-wide responses. Accordingly, we explore the potential of systems pharmacology therapeutics (SPTs) to address the multifactorial nature of BPD. As a prototype SPT, we describe the development of N-acetyl-L-lysyl-L-tyrosyl-L-cysteine amide (KYC), a systems chemico-pharmacology drug (SCPD), which is selectively activated in inflamed tissues and modulates key nodal targets such as high-mobility group box-1 (HMGB1) and Kelch-like ECH-associated protein-1 (Keap1). Collectively, the data suggest that future therapies may require a coordinated, network-level approach to break the destructive cycle and enable proper regeneration rather than partial repair.

## 1. Introduction

Bronchopulmonary dysplasia (BPD) is a multifactorial lung disorder frequently seen in premature infants. Classical BPD, first described by Northway et al. in 1967 [1], is a severe phenotype that was characterized by moderate prematurity (>30 weeks), severe lung damage, inflammation, and fibrosis resulting from prolonged exposure to high-concentration oxygen and prolonged mechanical ventilation. With the introduction of exogenous surfactant and the advancement of neonatal care, extremely premature neonates (<28 weeks) now have a much higher survival rate. Still, many require prolonged oxygen treatment and develop what is now termed “new BPD.” The pathologic changes of “new BPD” include alveolar simplification, large alveolar sacs with less extensive lung fibrosis and damage [2]. It is believed that the mechanism behind the new BPD is growth arrest [3] secondary to the impaired angiogenesis [4].

Respiratory distress syndrome resulting from a lack of surfactant formation used to be the primary cause of death in premature neonates. The introduction of exogenous surfactant [5] and advancements in neonatal care [6] have successfully increased the survival rate of premature neonates, including periviable (22–25 weeks) premature neonates [7]. Still, as a result, the prevalence of BPD has increased. Clinicians commonly define BPD as oxygen requirement at the postconceptional age of 36 weeks [8]. About half of the premature neonates born less at than 29 weeks of gestation are expected to develop BPD [9]. It is estimated that there are 10,000 to 15,000 new BPD cases diagnosed in the United States each year [10]. Postnatal steroid treatment used to be the standard treatment to prevent BPD under the belief that inflammation is the key contributor. However, the increased odds of neurodevelopmental impairment with early initiation [11] have resulted in a debate about its routine use in premature neonates [12].

Prolonged supplemental oxygen use, aggressive nutritional support, restricted fluid intake, systemic or airway steroids, and bronchodilators are commonly given to infants with established BPD. Although controversial, diuretics are also commonly included in the therapeutic regimen. With the increased work of breathing and concerns about aspiration, when and how to offer oral feeding to BPD infants remains a debated topic among neonatologists, dietitians, and speech therapists [13,14]. Each neonatal unit should establish its own feeding guidelines through a multidisciplinary discussion and drawing on best practices.

Postnatal vitamin A [15] and early postnatal caffeine treatment [16] are clinically proven beneficial treatments that can prevent premature neonates from developing BPD. Each treatment reduced BPD by about 10% in the randomized controlled study. Clinicians have not widely adopted the three-times-a-week intramuscular megadose vitamin A injection due to concerns about the lack of muscle mass in extremely premature neonates. The implementation of early postnatal caffeine and vitamin A treatments in premature neonates has not changed the prevalence of BPD [17]. Part of the reason for the unwavering prevalence of BPD is the improved survival rate in extremely premature neonates. This suggests that there is still a lack of clarity in our ability to treat such patients effectively.

Premature neonates frequently require supplemental oxygen to maintain adequate tissue oxygenation. However, the abrupt transition from a hypoxic (32–35 mmHg) intrauterine environment [18] to the ambient 21% oxygen (~150 mmHg) environment generates oxidative stress (OS) in their premature lungs. This development is especially challenging for extremely premature neonates, as they lack a fully developed antioxidative capacity and the capability to generate surfactant [19]. Although exogenous surfactant treatment improves oxygenation, surfactant does not contain an antioxidative property. Mechanical ventilation [20], antenatal inflammation [21], and postnatal infection [22] also contribute to the development of OS in premature lungs. Innate inflammatory cells, such as macrophages and neutrophils, recruited to the lung further exacerbate OS by releasing hypochlorous acid (HOCl), generated by myeloperoxidase (MPO) [23]. HOCl is a potent oxidant capable of damaging most biological molecules and cells [24].

OS and inflammation are often described as distinct pathophysiological components of BPD. However, they are functionally interconnected. Reactive oxygen species (ROS) not only damage lipids, DNA, and proteins but also activate pro-inflammatory transcription factors such as NF-κB and AP-1 [25,26]. In turn, inflammatory cytokines stimulate enzymatic ROS production through NADPH oxidase and myeloperoxidase, amplifying oxidative injury [27]. This bidirectional feedback loop drives a self-sustaining cycle of damage in the immature lung, complicating both mechanistic understanding and therapeutic targeting. As we recently reviewed in the context of BPD [28], the mechanistic interdependence between oxidative stress and inflammation undermines the efficacy of monotherapies that target only one of these pathways. Therapeutic approaches that isolate either oxidative stress or inflammation fail to interrupt the self-amplifying cycle that drives lung injury in BPD. [28].

**Figure 1:**
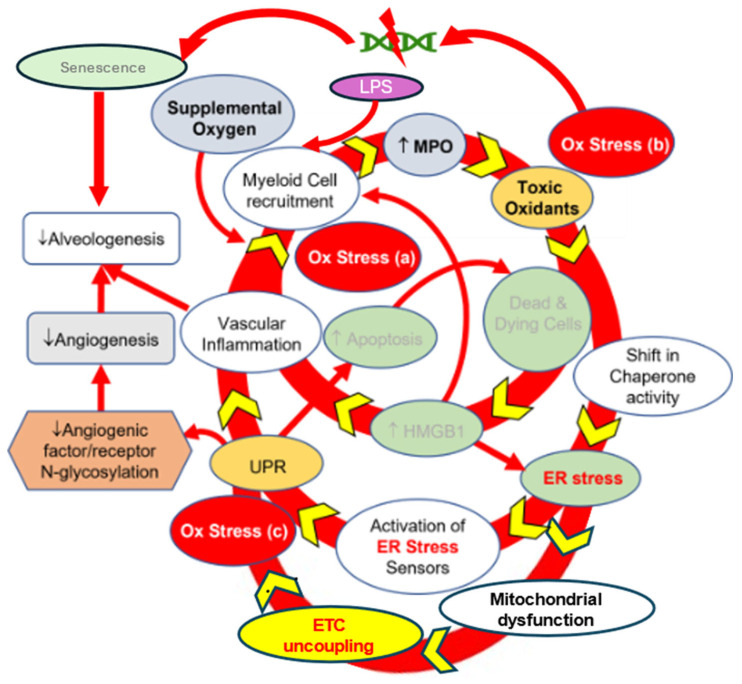
The proposed “destructive cycle” in the neonatal lung after exposure to supplemental oxygen. This figure focuses mainly on inflammation and oxidative stress (OS). Modified from [31].

The OS-injured cells release several endogenous molecules known as damage-associated molecular patterns (DAMPs). DAMPs bind to various pattern recognition receptors (PRRs) and exacerbate inflammation by activating the NF-κB transcription factor [29]. The mutual interaction between the OS and inflammation creates a complex, self-perpetuating, and destructive cycle (Figure 1) in the lungs of BPD patients [28]. The process is accompanied by increased endoplasmic reticulum (ER) stress, also referred to as the unfolded protein response (UPR), and mitochondrial dysfunction [27]. The protein refolding process or electron transport chain (ETC) uncoupling generates more reactive oxygen species (ROS), thereby further contributing to OS. After OS-induced lung injuries, the oxygen extraction capacity of the neonatal lung decreases due to fibrosis, alveolar simplification, and decreased alveolar blood vessel formation. Type 2 alveolar (AT2) epithelial cells, considered the resident progenitor cells [30], are reduced in the alveoli, resulting in an impaired growth trajectory [31].

The complex and self-reinforcing nature of these interconnected, pathological pathways requires a paradigm shift in designing effective therapeutic interventions for BPD. We propose that, instead of targeting isolated, individual pathways, the use of systems pharmacology therapeutics (SPTs) is a more appropriate approach. These SPTs act on multiple interrelated molecular targets within dysregulated biological networks. They differ from conventional multidrug regimens in that they can provide restoration of systems-level homeostasis through a single agent with multi-functional, network-directed activity [32,33]. For example, some SPDs are designed to target “nodes” or “motifs” within cellular circuits to rebalance signaling output [34]. The biological and computational logic behind this approach has been outlined in pioneering studies using disease networks and perturbation theory [35].

**Figure 2:**
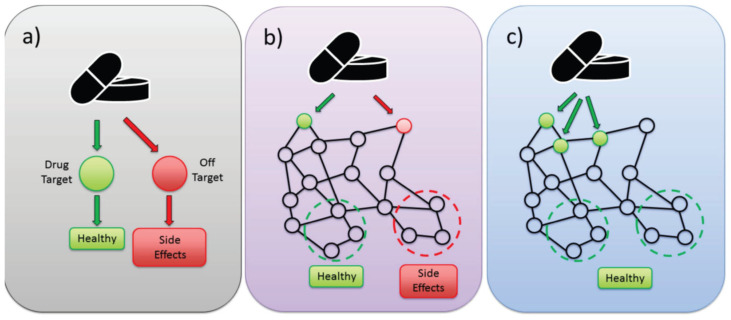
Comparison between classical drug design and systems pharmacology therapeutics (SPTs). (**a**) Representation of the one drug–one target model. (**b**) Representation of a systems biology perspective of therapeutic drug effects operating through a network. (**c**) Representation of the SPT one drug-multi-target-pathway/network approach of systems pharmacology. Green arrows: intended results; Red arrows: unwanted effects; dashed circles: final treatment outcomes.

We have captured and visualized this conceptual framework in Figure 2. The figure contrasts the traditional single-target drug model with the SPT paradigm. In the classical model, a drug binds to a single receptor or enzyme, blocking or activating its function, often disregarding the redundant, compensatory, or feedback-rich nature of biological systems. In contrast, SPTs engage multiple nodes, which are usually low-affinity or context-specific, and exert their effects through partial modulation of key network motifs, feedback loops, and signaling pathways. This distributed action can achieve greater therapeutic efficacy with reduced off-target toxicity. Importantly, SPT design does not necessarily require targeting multiple independent proteins but may instead involve multi-site engagement within a single regulatory network, restoring systems-level homeostasis [33]. This paradigm has been validated in complex adult disease conditions such as cancer and neurodegeneration, but has not been utilized in neonatology. We argue here that BPD is a quintessential systems disorder and SPT therapies are needed for efficacious treatment. We propose that this paradigm is particularly applicable to BPD and outline its potential in later sections of this review.

This review does not aim to provide an exhaustive inventory of mechanisms or clinical trials in BPD. Instead, it presents a focused synthesis of the complex and evolving pathobiology of BPD, integrating clinical insight with biological plausibility. The destructive cycle of oxidative stress and inflammation (Figure 1) provides a unifying framework for understanding disease onset, progression, and heterogeneity. Within this context, we introduce the concept of systems pharmacology therapeutics (SPTs) as a novel and integrative approach to drug development. By highlighting both the mechanistic rationale and translational challenges, we propose that SPTs may be particularly well-suited to disrupt the multifactorial cycles that drive BPD pathogenesis. As an illustrative example, we describe the emerging therapeutic profile of N-acetyl-L-lysyl-L-tyrosyl-L-cysteine amide (KYC), a redox-modulating peptide currently under investigation.

## 2. Signaling Pathways That Amplify the OS and Inflammation in BPD Lungs

Inflammation, OS, and mechanical injury are the three central contributors to the development and progression of BPD. Limited animal studies have been conducted on the mechanistic role of mechanical injury in BPD. In most animal studies, hyperoxia-induced OS and lipopolysaccharide (LPS)-induced inflammation are employed. However, the temporal relationship between OS and inflammation makes it difficult to discuss them separately [28].

### 2.1. Primary Mechanisms of Inflammation

There are two kinds of innate inflammation involved in BPD. The first is the inflammatory reaction to chorioamnionitis or nosocomial infection, probably in response to pathogens [28]. The second mechanism is through OS-activated residential macrophages, which attract neutrophils from the circulation to infiltrate the damaged site, resulting in sterile inflammation [36,37]. Regardless of the mechanism, neutrophils release molecules to remove pathogens or dead or dying cells after arriving in the alveoli. The activated macrophages and neutrophils generate superoxide (O_2_^•−^) or hydrogen peroxide (H_2_O_2_) through NADPH-oxidases (NOXs). O_2_^•−^ interacts non-enzymatically with nitric oxide (•NO) released by the inducible NO synthase (iNOS or NOS2) to form a potent reactive nitrogen species—peroxynitrite (ONOO^−^) [38]. H_2_O_2_ interacts with the nearby chloride anion to generate a potent ROS—HOCl—enzymatically by the MPO released from the activated myeloid cells [39]. Other halides and pseudohalides can also form ROS by the exact mechanism. Both ONOO^−^ and HOCl are highly reactive oxidants that contribute significantly to oxidative injury and cellular dysfunction in the neonatal lung.

The sterile and pathogen-driven inflammatory mechanisms described above are functionally intertwined with OS via a shared signaling architecture involving MPO, NOXs, and cytokine-driven feedback. From a systems pharmacology perspective, this suggests that modulating a single node, such as MPO or NF-κB, may lead to partial or transient benefits unless broader network regulation is achieved. SPTs are designed to simultaneously target multiple redox and inflammatory regulators, exemplifying this systems-level intervention (see Section 4.2 below).

#### 2.1.1. DAMPs and PRRs

The activated neutrophils release their content to facilitate the removal of pathogens or injured cells, mainly through forming free radicals or releasing proteases. The wounded, dying, or dead cells, including neutrophils, release their contents as damage-associated molecular patterns (DAMPs) [40]. The most extensively studied DAMP molecule is high-mobility group box 1 (HMGB1). HMGB1 is a nuclear protein that helps maintain the structure and function of chromosomes [41]. After being released from the nucleus, HMGB1 undergoes post-translational modifications, including oxidation, phosphorylation, *S*-nitrosylation, lactylation, and acetylation [42]. Both lactylation and acetylation promote the release of HMGB1 into the cytosol [43], which is the first step in its extracellular release.

An increased level of HMGB1 has been reported in tracheal aspirates [44] and serum [45] of premature infants who later developed BPD. In a mouse BPD model, the administration of an HMGB1 fragment that competitively inhibits HMGB1 successfully ameliorated the severity of BPD [46]. HMGB1 contains three thiol groups; their redox state determines the biological function of HMGB1. The fully reduced form of HMGB1 is pro-inflammatory but may accelerate tissue regeneration by activating the quiescent stem cells [47]. The fully oxidized form of HMGB1 is generally considered anti-inflammatory and does not promote chemotaxis [48]. Modulating the redox state of HMGB1 has thus been thought to have therapeutic potential in modulating inflammation [49].

**Figure 3:**
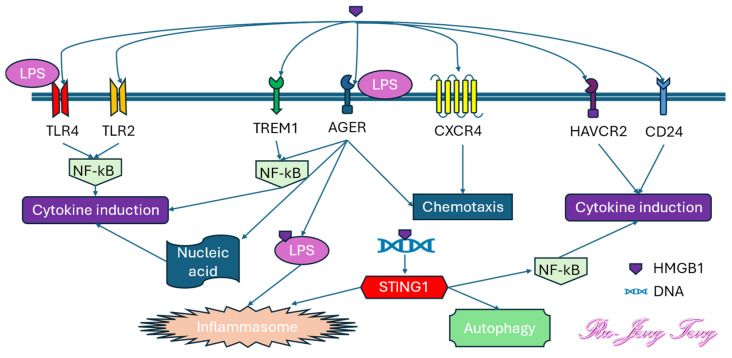
High-mobility group box-1 (HMGB1) is the most potent damage-associated molecular pattern (DAMP) family molecule. The binding between HMGB1 and pattern recognition receptors (PRRs) activates several downstream signaling pathways, exacerbating inflammation by enhancing inflammatory cell chemotaxis, cytokine release, and inflammasome formation. NF-kB is the primary mediator for cytokine induction. STING1 (Stimulator of Interferon Response cGAMP Interactor 1 or Stimulator of Interferon Genes 1) is an adaptor protein that promotes inflammasome formation.

Extracellular HMGB1 can bind to several pattern recognition receptors (PRRs) on the cell membrane, including Toll-like receptors 2 and 4 (TLR2 and TLR4), advanced glycosylation end-product specific receptor (AGER), triggering receptor expressed on myeloid cells 1 (TREM1), C-X-C motif chemokine receptor 4 (CXCR4), cluster of differentiation 24 (CD24), and hepatitis A virus cellular receptor 2 (HAVCR2) [50]. The binding between HMGB1 and TLRs, AGER, or TREM1 activates the NF-kB signaling pathway and induces the formation of inflammatory cytokines. HMGB1 also facilitates the binding between CXCL12 and CXCR4, promoting the chemotaxis of inflammatory cells (Figure 3).

Other DAMP molecules, including extracellular DNA [51], IL1α [52], IL33 [53], cyclophilin A [54], HSP70 [55], and S100 [56] have also been implicated in the development of BPD. However, their mechanistic roles remain incompletely explored.

#### 2.1.2. Inflammasome Pathway

**Figure 4:**
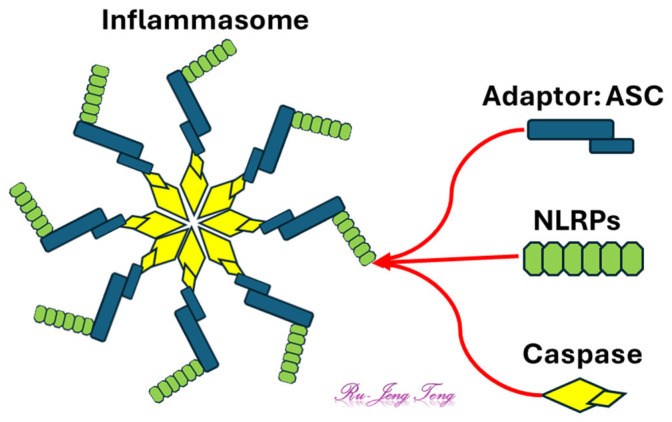
Structure of the inflammasome. NLRP (receptor), caspase (effector), and adaptor are the three main components of the inflammasomes. Some of the inflammasomes do not contain the adaptor.

Inflammasomes are intracellular protein complexes comprising caspases, sensors, and adaptors (Figure 4). Inflammasomes cleave pro-inflammatory cytokines into active forms [57]. The caspases, especially caspase-1, induce apoptosis or pyroptosis, which is critical to releasing inflammatory mediators [58]. Caspases involved in the inflammasome formation include the canonical caspase-1, the non-canonical caspase-11 (also known as caspase-4 or caspase-5 in humans), or caspase-8. PYCARD, or ASC, is the adaptor protein for most inflammasomes. The primary sensors are the NOD-like receptors (NLRs), including NLRP3, NLRP1, NLRC4, and AIM1. Pyrin also serves as a sensor for the inflammasome [59,60]. The NLRP3 inflammasome is crucial for sterile inflammation [61]. After detecting specific DAMPs or pathogen-associated molecular patterns (PAMPs), the sensor will either oligomerize or interact with other proteins to form the inflammasome.

The inflammasome pathway and DAMPs are closely related. In response to DAMPs, DNA from damaged cells is converted into cyclic guanosine monophosphate–adenosine monophosphate (cGAMP) by the cyclic GMP-AMP synthase (CGAS). Stimulator of interferon genes 1 (STING1) acts as a receptor for cGAMP, producing type I interferons and subsequent inflammasome formation [62]. DAMPs can also activate the inflammasome directly, especially the NLRP3 inflammasome, releasing IL-1β, IL-18, and other pro-inflammatory cytokines [63]. Gasdermin D (GSDMD) is the key executor, which is cleaved by caspase-1/-4/-5/-11, upon inflammasome activation, into the N-terminal fragment (GSDMD-NT) [64]. GSDMD-NT oligomerizes to form a pore in the cell membrane, releasing inflammatory cytokines and causing pyroptosis [65]. The released cytokines then augment inflammation by recruiting more inflammatory cells.

The inflammasome represents a central “hub” within the inflammatory signaling network, influenced by both oxidative inputs and DAMP signaling (Figure 4). Systems pharmacology therapeutics that dampen upstream triggers (e.g., MPO-generated HOCl) and modulate redox-sensitive proteins (e.g., thiolated HMGB1) could disrupt inflammasome activation at multiple control points. This reinforces the logic of targeting interconnected pathways rather than isolated effectors.

### 2.2. Uncoupled Endothelial Nitric Oxide Synthase (eNOS)

NO generated by the endothelial nitric oxide synthase (eNOS or NOS3) plays a critical role in vascular endothelial cell growth factor (VEGF)-induced angiogenesis [66]. •NO also modulates mitochondrial biogenesis [67], which is essential for endothelial cell function. Proper eNOS activity requires phosphorylation, dimerization, interaction with heat-shock protein 90, and many cofactors, including tetrahydrobiopterin (BH4) [68]. The absence of any cofactor can lead to eNOS uncoupling, generating superoxide instead of •NO [69] and mitochondrial dysfunction [70]. GTP-cyclohydrolase 1 (GCH1) is the critical enzyme in the BH4 biosynthesis. We have reported decreased GCH1 enzyme activity and BH4 levels in BPD rat lungs [71]. The uncoupled eNOS and subsequent mitochondrial dysfunction contribute to the increased OS in BPD lungs. Superoxide generated by the uncoupled eNOS interacts with the limited amount of •NO at a rate constant of 6.7 ± 0.9 × 10^9^/mol/s to form the potent ONOO^−^ [72] that oxidizes almost all biological molecules.

eNOS also regulates neutrophil functions through •NO formation [73]. The basal level of •NO limits neutrophil chemotaxis by reducing the CXCR2 expression [74], prevents the circulating neutrophils from adhering to the activated endothelial cells, suppresses neutrophil phagocytic activity, modulates cellular 5-lipoxygenase activity and leukotriene synthesis, and enhances the cell death mechanism of the infiltrated neutrophils [75]. Thus, disrupted basal eNOS activity may facilitate neutrophil-mediated tissue damage.

### 2.3. Endoplasmic Reticulum (ER) Stress or Unfolded Protein Response (UPR)

The ER is the subcellular organelle where proteins are sequentially folded and modified to reach their full function. Post-translational protein glycosylation is either accomplished or initiated in the ER. It is also a site for lipid and cholesterol synthesis, calcium storage, and detoxification [27]. The ER also intimately interacts with other organelles, especially the neighboring mitochondria, to support their function. The neonatal lung responds to hyperoxia-induced OS acutely with an ER stress response as a coping mechanism [23]. This response reserves critical biomolecules to increase the chance of cell survival. However, ER stress can also cause translational attenuation, inflammation, autophagy, apoptosis, mRNA and protein degradation, and increase OS when the exogenous stress is insurmountable [27]. Although ER stress resolves quickly after OS is removed [23], its persistence in the early postnatal life still impairs the lung growth trajectory [76].

**Figure 5:**
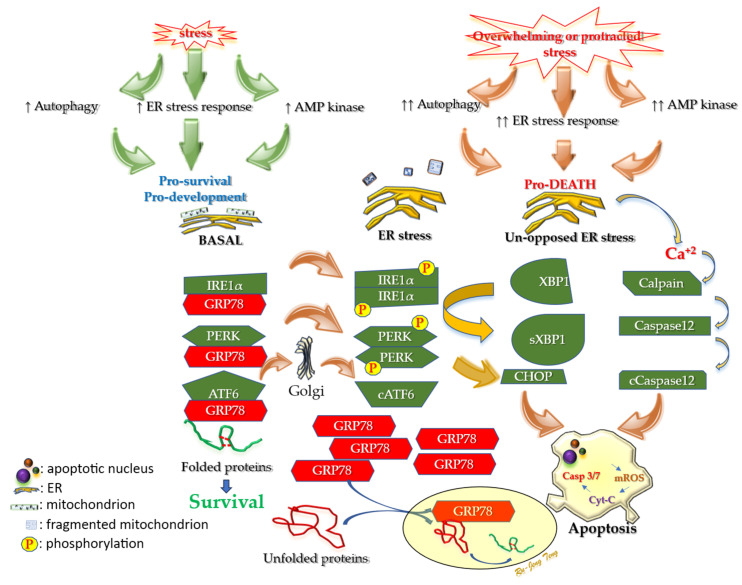
Schematic summary of endoplasmic reticulum (ER) stress or unfolded protein response (UPR). Stress distorting correct protein folding in the ER can elicit the ER stress response. Basal ER stress upregulates the synthesis of the endogenous chaperone (BiP/GRP78) that assists protein refolding. Basal ER stress also inhibits the synthesis of non-essential proteins or degrades them so that raw material can be generated to synthesize essential proteins. Basal ER stress response is a survival mechanism for cells (**left panel**). If, however, the stress is overwhelming, BiP/GRP78 will all leave the ER stress sensors (IRE1α, PERK, and ATF6) to cope with the unfolded proteins. The protein refolding process generates ROS that exacerbate OS and lead to cell death. AMP: adenosine monophosphate; BiP: binding immunoglobulin protein; GRP78: glucose-regulated protein 78; IRE1α: inositol-requiring enzyme 1α; PERK: protein kinase R-like ER kinase; ATF6: activating transcription factor 6; cATF6: cleaved ATF6; mROS: ROS from mitochondria; Cyt-C: cytochrome C; Casp 3/7: caspase 3 and 7; CHOP: C/EBP homologous protein; XBP1: X-box binding protein 1 (XBP1); sXBP1: split XBP1. (The figure is reproduced from [27] under the Creative Commons CC BY 4.0 license).

In the endoplasmic reticulum (ER), there is a group of protein chaperone ER stress sensors, including IRE1α, PERK, and ATF6. These endogenous chaperones include BiP (also known as GRP78), GRP94, CRT, and PDI. BiP is the most extensively investigated chaperone protein. Any exogenous stress that disrupts protein folding (proteostasis perturbation or proteotoxicity) can encourage GRP78 to abandon the ER stress sensors, allowing them to be activated either through phosphorylation and dimer formation (IRE1α and PERK), or be translocated to the Golgi apparatus for activation through enzymatic cleavage (ATF6) (Figure 5). The liberated GRP78 can thus help nascent proteins to be refolded appropriately. Protein folding is a redox process that relies on PDI and ERO1. H_2_O_2_ is a byproduct of the protein folding in the ER that requires glutathione peroxidases and peroxiredoxin 4 to remove it. The excessive protein refolding will thus exacerbate ER stress, leading to increased OS. The disrupted ER-mitochondria interaction during ER stress also causes mitochondrial dysfunction [77], with an uncoupled ETC [78], contributing to OS.

All three ER stress sensors participate in the inflammatory response [79]. IRE1α and PERK activation lead to NF-κB-induced inflammatory response [80]. ATF6 cleavage activates NF-κB through the PI3K-Akt pathway [81]. HMGB1 is released from autophagic flux-induced cell death under ER stress, contributing to another mechanism for sterile inflammation [82]. Many signaling pathways are involved in the ER stress-induced inflammation. The phosphorylation and dimerization of IRE1α and PERK activate downstream NF-kB [83] and MAPK [84] signaling pathways, subsequently producing inflammatory cytokines. ER stress also activates the NLRP3 inflammasome, releasing IL-1α to elicit sterile inflammation [85]. Through NF-kB, JNK, and IRF3 pathways, ER stress hyperactivates neutrophils by forming neutrophil extracellular traps (NETosis) [86,87]. Undoubtedly, ER stress-mediated inflammation also contributes to OS. The reciprocal relationship between ER stress and oxidative damage highlights the importance of targeting systems at a higher level. The restoration of ER homeostasis by SPTs, such as through NRF2 activation and modulation of redox-regulated chaperones, may afford dual benefits in reducing inflammation and preserving alveolar development.

### 2.4. Cellular Senescence

The unopposed OS encountered by neonatal lungs, especially premature lungs, can damage the DNA of lung cells. To avoid faulty replication, tumor suppressor genes are activated to arrest the cell cycle. When cells enter irreversible growth arrest, it is referred to as cellular senescence [88]. However, it is now clear that cellular senescence might not be an irreversible change [89]. There are multiple types of cellular senescence, such as replicative senescence, oncogene-induced senescence, therapy-induced senescence, and developmental senescence [90]. Senescent cells are usually larger and metabolically active, but prone to using glycolysis instead of oxidative phosphorylation in ATP formation, even in a well-oxygenated environment [91]. Their peculiar metabolic behavior inhibits the growth of surrounding cells by depriving nutrients and energy. Senescent cells exhibit a characteristic senescence-associated secretory phenotype (SASP), actively secreting cytokines, growth factors, and proteases [92]. SASP has a paracrine effect that can transform neighboring cells into a senescent state [93].

Developmental senescence plays a critical yet essential role during fetal organ development [94]. This explains why the percentage of senescent cells is high in the mouse lung immediately after birth and decreases to the nadir at the beginning of the alveolar stage [94]. Acute senescence is recognized as a critical biological adaptation in wound healing, as it releases growth factors [95]. To resolve acute senescence, phagocytes remove senescent cells. If the senescent change is too extensive or becomes chronic, it will impair the function and growth of the organ. This is especially detrimental to premature organs, as the growth trajectory will be impaired. We have reported increased cellular senescence in human and rat BPD lungs. The senescent change in AT2 and endothelial cells indicates its contribution to BPD [31].

Senescent cells often exhibit senescence-associated mitochondrial dysfunction (SAMD), which includes increased NOX activity, impaired antioxidant defenses, and uncoupled ETC activity that increases ROS formation [96]. No specific cytokine is consistently present in the SASP. However, IL-1, IL-6, IL-8, IL-18, TNFα [97], and HMGB1 [98] are frequently described in the literature. These pro-inflammatory mediators support a chronic inflammatory milieu and promote fibrosis. Through persistent cytokine release, SASP factors create a pro-inflammatory microenvironment that reinforces tissue injury, impairs repair, and promotes fibrotic remodeling—principal features of the BPD phenotype.

### 2.5. Metabolic Disruption

Metabolic dysregulation has been observed in BPD [99]. The disturbed metabolism includes glucose, free fatty acids, and amino acids. Ratner et al. were the first group to describe an association between impaired mitochondrial complex I activity and alveolar simplification in mouse pups after a short exposure to hyperoxia, suggesting an essential role of oxidative phosphorylation in alveolar formation [100]. Gong et al. observed an increased glycolysis and pentose phosphate pathway (PPP) in hyperoxia-exposed mouse pups and lung endothelial cells. In their report, the increased PPP, not glycolysis, results in abnormal endothelial cell proliferation, dysmorphic angiogenesis, and alveolar simplification [101]. In the rat BPD model, hyperoxia exposure resulted in an enrichment of glycolysis, oxidative phosphorylation, and PPPs by Affymetrix transcriptomic analysis, but a paradoxical decrease in oxidative phosphorylation (manuscript submitted), probably due to ER stress and mitochondrial fragmentation [76]. Targeted metabolomics in the same study revealed an increased glucose transport into the BPD lungs, without a corresponding increase in ATP generation, suggesting impaired oxidative phosphorylation. The changes in acylcarnitines and amino acids also suggest an uncoupled ETC.

## 3. Signaling Pathways That Impair Angiogenesis in BPD Lungs

Impaired angiogenesis is believed to play a central role in BPD development and progression [4]. Endothelial cells can be classified into tip and stalk cells during sprouting angiogenesis [102]. However, this classification does not apply to alveolar formation. Alveolar capillary endothelial cells are classified into aerocyte (aCap) and general capillary endothelial cell (gCap) [103]. Some gCap cells are in the proliferating stage and can be classified as “proliferating gCap.” Some consider proliferating gCap cells the local stem-like cells that orchestrate microvascular repair after injury [104]. Although these lung endothelial cells rely mainly on glycolysis, impaired oxidative phosphorylation can impair the proliferation, resulting in alveolar simplification. We describe a few signaling pathways that have been studied.

### 3.1. eNOS Uncoupling

The eNOS-generated NO is crucial for successful angiogenesis [105] as it serves as the second messenger of VEGF and modulates the mitochondrial function of the endothelial cells [106]. It was demonstrated that eNOS is uncoupled in the rat BPD model, partly due to the BH4 deficiency [71]. The uncoupled eNOS and mitochondrial dysfunction thus contribute to OS and impair angiogenesis. The insufficient •NO formation after eNOS uncoupling cannot adequately mediate the VEGF signaling. The •NO deficiency will not be able to remove the superoxide generated by the complex I or to support mitochondrial biogenesis. eNOS uncoupling illustrates how a single dysfunctional enzymatic process can simultaneously impair angiogenesis, increase oxidative stress, and alter the behavior of immune cells. Therapeutics that restore eNOS coupling via cofactor preservation, redox modulation, or upstream antioxidant signaling could address multiple axes of BPD pathology. This is a hallmark of systems pharmacology strategies.

### 3.2. ER Stress

ER stress impairs angiogenesis by at least two mechanisms. Multiple growth factors, secretory and transmembrane proteins, acquire various post-translational modifications for different biological purposes. N-Glycosylation is one of the post-translational modifications that is initiated in the ER. A characteristic example is the VEGF receptor 2 (VEGFR2) in the ER [107]. Increased ER stress has been demonstrated in the hyperoxia rat BPD models [23,76], and decreased VEGFR2 glycosylation was also seen in this model. Treatment with tunicamycin, a characteristic ER stress inducer, also supports the contribution of ER stress to BPD, which resulted in a BPD phenotype with alveolar simplification and decreased blood vessel count. The ability of tauroursodeoxycholic acid, a known chemical chaperone that reduces ER stress, to attenuate BPD severity induced by hyperoxia and tunicamycin undoubtedly provides further evidence of the role of ER stress in BPD [76]. The intimate interaction between the ER and mitochondria is another mechanism by which ER stress impacts angiogenesis. The uncoupled electron transport chain resulting from the ER stress can lead to pathological angiogenesis [108] and impair tip cell function during the sprouting angiogenesis [109] as described in the next section.

### 3.3. Mitochondrial Dysfunction

The lungs generate a significant amount of lactic acid, primarily due to the high percentage of endothelial cells [110]. Although endothelial cells rely mainly on aerobic glycolysis to provide energy [109], they require both glycolysis and oxidative phosphorylation for proliferation [111]. The impaired complex I activity in hyperoxia-exposed neonatal lungs impairs angiogenesis and increases OS. Gong et al. reported that hyperoxia causes dysmorphic angiogenesis [101], which is associated with disrupted mitochondrial metabolism of glucose, free fatty acids, and amino acids. We did not observe dysmorphic angiogenesis, but we did notice a decrease in the percentages of aerocytes and proliferating general capillary endothelial cells, indicating a reduced ability to form new blood vessels (manuscript submitted).

### 3.4. Cellular Senescence

The relationship between cellular senescence and angiogenesis is a complex one. This complexity comes from different types of senescence and the SASP. In certain situations, senescent cells can promote angiogenesis. This can be observed in the pathologic neovascularization associated with diabetic retinopathy [112]. However, cellular senescence can disrupt the function of endothelial cells, especially progenitor cells, and the response to angiogenic stimuli [113]. Decreased VEGF expression could be the reason behind radiation-induced endothelial cell senescence [114], whereas YAP1 signaling has been considered the cause behind aging-related endothelial cell senescence [115].

We have reported increased senescent changes in endothelial and AT2 cells in rat BPD lungs [31]. Multiple putative signaling pathways are involved in this process, including ER stress, autophagy, TGFβ/SMAD, PTEN/PI3K, AMPK, PGC1α, etc. [94]. These signaling pathways interact with each other and ultimately lead to impaired angiogenesis. The improved alveolar complexity and blood vessel count by foxo4-dri, a synthetic senolytic agent [116], provide further evidence that cellular senescence contributes to the impaired angiogenesis in BPD.

Given the convergence of the multiple pathways in BPD, such as oxidative stress, ER stress, senescence, and impaired angiogenesis, on common molecular regulators, a therapeutic capable of modulating several of these simultaneously could provide superior clinical benefit. The concept of systems pharmacology is particularly compelling in this context: by partially correcting imbalances across a network. SPTs should help restore developmental trajectories more effectively than monotherapies.

## 4. Therapies for BPD: From One-Target Drugs to Systems Pharmacology

### 4.1. Historical and Current One Target–One Drug Paradigm

Drug discovery and development in neonatology remain limited and poorly aligned with the unique physiological and maturational trajectories of premature infants [117,118]. In particular, over the past three decades, multiple therapeutic interventions have been tested to reduce the incidence and severity of BPD. Many of these therapies were not initially developed or rigorously tested for neonates, but rather repurposed from adult medicine and used off-label. Most target individual aspects of disease pathophysiology, such as inflammation, oxidative stress, or respiratory drive, reflecting the traditional “one drug–one target” paradigm [28]. While some have demonstrated short-term physiological benefits, their overall impact on long-term pulmonary outcomes has been limited. Moreover, none of these interventions consistently disrupt the self-reinforcing pathological cycle that drives disease progression. In the following sections, we highlight several major therapeutic strategies, including corticosteroids, caffeine, vitamin A, and stem cell therapies and evaluate their benefits, limitations, and implications for future treatment approaches.

#### 4.1.1. Steroids

Although the American Academy of Pediatrics does not recommend it due to the potential complication of neurodevelopmental deficit, some clinicians still use early systemic steroids to prevent or treat BPD in a selective group of premature neonates at high risk [119]. Steroids’ main biological effect stems from their non-selective anti-inflammatory properties. Steroids do not have antioxidant activity themselves. The antioxidant property of steroids is believed to be a secondary effect through upregulating certain antioxidant enzymes.

#### 4.1.2. Vitamin A

Vitamin A has anti-inflammatory and antioxidant properties. The hydrophobic chain of polyene units of vitamin A can quench singlet oxygen, neutralize thiyl radicals, and stabilize peroxyl radicals [120]. However, this antioxidant property only occurs in a low-oxygen tension environment. Vitamin A will auto-oxidize when the oxygen tension is high. The anti-inflammatory property has been demonstrated in several randomized controlled studies, using inflammatory markers as the primary endpoint [121]. So far, only intramuscular high-dose injections have been beneficial. Meta-analysis of the four available randomized controlled studies using enteral low-dose vitamin A failed to show efficacy [122]. The inhaled vitamin A study is presently ongoing.

#### 4.1.3. Caffeine

Caffeine is probably one of the most extensively studied medications in neonatology. The primary hypothesis of the CAP study was that a better neurological outcome would be achieved by reducing apnea of prematurity. Interestingly, the successful reduction in the rate of BPD, one of the secondary outcomes, has led to almost universal implementation among clinicians [16]. Our group has demonstrated in the rat BPD model that caffeine increases BH4 levels and eNOS phosphorylation [71], decreasing neutrophil infiltration, apoptosis, and ER stress [23], which may explain the improved angiogenesis and alveolar formation. BH4 is also an antioxidant. Endesfelder et al. demonstrated the antioxidative effect of caffeine by showing decreased H_2_O_2_, malondialdehyde, and 8-hydroxy-deoxyguanosine with increased expression of superoxide dismutases in the hyperoxia rat BPD model [123]. They also showed caffeine’s anti-inflammatory activity by decreasing NF-kB expression using the same animal model [124]. Köroğlu et al. used the rat intra-amniotic lipopolysaccharide injection BPD model to show a reduced inflammatory response with improved lung morphometry and respiratory system resistance by caffeine treatment [125]. Other potential benefits of caffeine treatment include antifibrotic and diuretic effects [126]. Caffeine protects the neonatal lung against the development of BPD through multiple mechanisms.

#### 4.1.4. Stem Cell Therapy

Stem cells [127] and stem cell-derived extracellular vesicles [128] treatments are promising for BPD. Phase I [129,130] and II clinical studies (NCT03601416) are ongoing. Mesenchymal stem cells (MSCs) are the most used source due to their ease of isolation, low immune reaction, anti-inflammatory and antioxidative properties [131,132], and reparative capabilities [127,133]. MSC secretome [134] or exosome [135] are new stem cell-based modalities. The available short-term results of the phase I trials showed that premature neonates tolerated stem cell therapy well without apparent side effects [130]. Still, concerns regarding the difficulty in obtaining the appropriate number of cells and maintaining their quality, as well as the potential for vascular occlusion and tumorigenicity, remain unresolved [136].

These traditional therapeutic strategies for BPD, while biologically grounded and sometimes clinically useful, have not produced consistent or transformative outcomes. This reflects the limitations of targeting isolated disease mechanisms in a condition driven by a complex, interconnected network of oxidative stress, inflammation, and disrupted development. These shortcomings underscore the need for a paradigm shift toward systems-level therapeutics that can address the multifactorial nature of BPD pathogenesis.

### 4.2. Systems Pharmacology Therapeutics: A New Paradigm for Complex Diseases

#### 4.2.1. Need for a New Therapeutic Framework

The failure of traditional BPD therapies to consistently improve long-term outcomes reflects more than incremental inefficiency. It also underscores a deeper conceptual limitation of the prevailing pharmacologic approach. For decades, drug development has been guided by the “one drug–one target–one disease” paradigm, which has proven effective in treating conditions with discrete, well-defined etiologies, such as bacterial infections or certain forms of cancer [33,35]. However, this reductionist model is poorly suited for treating complex, multifactorial diseases like BPD. As noted above, a myriad of biological processes, such as interdependent OS associated with inflammation, ER stress, senescence, and disrupted lung development, are mechanistically entangled and mutually reinforcing [27,28,137]

The need for a systems-level therapeutic strategy is especially compelling in BPD, where disease drivers exhibit bidirectional feedback, redundancy, and compensation. In such settings, inhibiting a single node often triggers adaptations in adjacent or upstream pathways, leading to limited efficacy or off-target consequences. Thus, a new framework is required that can modulate multiple disease-relevant nodes while accounting for feedback, crosstalk, and nonlinear system responses. This is the conceptual foundation of SPTs. Such therapeutic drugs are an emerging class of interventions designed to address disease as an integrated network rather than a linear sequence of isolated events [138,139].

#### 4.2.2. Definition of an SPT

SPTs are not simply multitarget drugs. Instead, they are intentionally designed or selected to engage multiple nodes within a pathological network in a coordinated, system-aware fashion [33,35,140]. The goal is to restore or reprogram system-level behavior rather than merely suppress an isolated molecular signal.

Historically, the roots of systems pharmacology date back to the 1940s, when early frameworks sought to encompass the complexity and variability of human physiology and pathology. The advent of high-throughput multiomics, network analysis, and computational modeling in the 1990s and 2000s helped crystallize systems biology into a practical foundation for drug design [140]. Talevi later introduced the concept of the “master key” therapeutic capable of unlocking multiple related nodes within a disease network, contrasting with the conventional “lock-and-key” model [141].

Essential features of SPTs include the following:

- Multinodal Engagement: Simultaneous modulation of multiple interrelated targets within the same biological circuit.
- Modulatory (Not Ablative) Action: SPTs often tune rather than eliminate function, favoring homeostatic correction over complete suppression.
- Network-Driven Outcomes: Effects emerge from interaction dynamics across the system, not simple target summation.
- Emergent System Reprogramming: This may include the activation of adaptive responses, such as NRF2, the restoration of mitochondrial balance, or the dampening of DAMP signaling.

To further clarify what constitutes a true systems pharmacology therapeutic, it is useful to examine drugs that, while biologically broad in action, lack the design coherence and network specificity that define SPTs. For example, aspirin is often proposed as an SPT. Indeed, it has been demonstrated to be a pleiotropic agent, binding over two dozen targets including COX-1/2, NF-κB, and numerous acetylation-sensitive proteins [33]. It also exhibits diverse biological effects such as anti-inflammatory, anti-thrombotic, and chemopreventive actions, yet it is not an SPT.

Aspirin’s broad actions are the result of biochemical promiscuity rather than rational network targeting. Its effects are neither coordinated nor intentionally designed to modulate disease networks in a systemic, balanced manner. Moreover, its lack of targeted coherence can produce harmful side effects, such as GI bleeding and renal stress. This distinction highlights a fundamental principle: multi-targeting does not equate to systems pharmacology. What defines an SPT is not the number of targets, but their intentional engagement as part of a coherent, disease-relevant network strategy. True SPTs stand at the intersection of systems biology, network pharmacology, and translational medicine, offering a next-generation solution for diseases where linear pharmacology has reached its practical and efficacious limits [139,142].

#### 4.2.3. BPD Complexity and SPTs

BPD epitomizes a systems-level disease. Its progression involves recursive feedback among oxidative stress, inflammation, ER stress, and disrupted alveolar and vascular development [26]. Each of these domains reinforces the others, creating a self-sustaining pathophysiological cycle. Figure 1 illustrates this loop: ROS generation enhances cytokine production, which in turn promotes ER stress and senescence, thereby exacerbating oxidative injury. Furthermore, standard interventions such as corticosteroids or vitamin A act on individual elements of this system and thus do not break this destructive cycle. Redundancy in cytokine signaling, compensatory metabolic flux, and nonlinear amplification all conspire to make one-target therapies ineffective or transient in their benefits.

An ideal BPD-directed SPT should

- Scavenge ROS while modulating MPO activity.
- Activate NRF2-driven antioxidant gene expression.
- Reduce pro-inflammatory cytokine signaling in specific immune cell subsets.
- Protect epithelial and endothelial progenitor cell pools critical for lung development.

Such coordinated actions cannot be achieved through drug cocktails or traditional multitarget drugs. Instead, they require system-aware molecules designed to engage the disease network holistically.

#### 4.2.4. Strategic Considerations in Developing SPTs

Developing SPTs presents unique challenges but holds the potential to transform care for complex disorders like BPD. Critical development principles include the following:

- Network-Informed Targeting: Use of transcriptomic/proteomic data to map disease-relevant subnetworks.
- Phenotypic Screening: Prioritize functional outcomes in disease models over single-target readouts.
- Pharmacodynamic Complexity: Recognize that efficacy may reflect delayed or emergent system changes, requiring longitudinal biomarker strategies [143].
- Safety Profiling at the Network Level: Systems toxicology is essential to predict effects on unintended but connected pathways [138].

Despite the conceptual maturity of systems pharmacology in adult diseases, neonatology remains largely untouched by these frameworks. No published clinical or preclinical studies, to our knowledge, have purposefully applied SPT principles to BPD. This highlights both the novelty and the opportunity of doing so. Our group has been developing an end-capped tripeptide that exemplifies the SPT model, targeting MPO catalysis to generate thiyl radicals that modulate protein networks, and activating NRF2 while reducing HMGB1 release and senescence markers. It demonstrates how intentional molecular design can generate systems-level therapeutic outcomes, and this is discussed in more detail in Section 4.3 below.

### 4.3. KYC as an SPT

#### 4.3.1. What Is KYC?

KYC is a synthetic end-capped tripeptide originally developed to intercept inflammatory oxidants in vivo. It is now recognized as a prototype of a novel drug class known as systems chemico-pharmacology drugs (SCPDs). SCPDs are not defined solely by their target profile, but rather by their chemical reactivity, context-dependent activation, and network-level pharmacodynamics. As described in our patent [144], an SCPD interacts with a “first target”, which is often a catalytically active enzyme such as myeloperoxidase (MPO). The latter is then modified chemically, such as via oxidation, to form the parent compound (KYC). This activation step creates a reactive intermediate (e.g., a thiyl radical) capable of selectively engaging with other nearby biomolecules, which are referred to as the “second target(s)” within the pathological microenvironment. Thus, an SCPD uses a two-step, context-locked mechanism: the first interaction activates the parent molecule, and the second extends its pharmacological sphere of influence across the surrounding system.

This molecular choreography provides both spatial specificity (as activation occurs only in disease-associated biochemical zones) and network coherence, enabling the SCPD to reshape aberrant biological signaling in a systemic yet controlled fashion. KYC embodies this principle, since it becomes pharmacologically active only within inflamed tissues rich in MPO, and its subsequent thiylation of key signaling proteins promotes a homeostatic rebalancing rather than toxic ablation. As such, this SCPD offers a next-generation strategy for treating complex, nonlinear diseases like BPD.

#### 4.3.2. Mechanism of Action: From MPO Activation to Site-Specific Network Modulation

KYC operates through a unique multi-step redox mechanism that defines its systems-level pharmacology:

- Myeloperoxidase (MPO) Activation: KYC is selectively activated at sites of inflammation by MPO, a heme-containing enzyme released by neutrophils. MPO catalyzes the oxidation of the tyrosine residue in KYC, forming an initial tyrosyl radical and then, via intramolecular electron transfer, converting the cysteine thiol into a thiyl radical. This activation is contingent on MPO’s catalytic cycle and thus restricted to inflammatory microzones.
- Sphere of Influence and Local Specificity: Once formed, the thiyl radical exits the MPO catalytic site and engages proximal protein thiols or oxidized amino acids, enabling reversible, site-specific covalent modification. We have termed this concept the “sphere of influence,” which confers selectivity by limiting KYC’s reactive behavior to a defined microenvironment shaped by inflammatory enzyme release, pH, and redox potential. In addition, the short-lived thiyl radical ensures both spatial precision and temporal constraint, limiting off-target effects. Unlike classical inhibitors, KYC does not irreversibly bind to targets but instead modifies them through thiolation, enabling the reprogramming of key signaling hubs, such as HMGB1, GSNOR, Keap1, and NF-κB regulators.
- Emergent Network Effects: These covalent modifications reset cellular redox tone, activate NRF2 signaling, dampen DAMP-associated inflammation, and restore mitochondrial homeostasis. KYC thus engages multiple, interlinked targets as part of a broader strategy to reestablish physiological equilibrium.

This chemical mechanism of KYC satisfies several defining criteria for SPTs outlined in Section 4.2, including multinodal engagement, homeostatic tuning, and emergent reprogramming of complex inflammatory networks. Taken together, these findings not only validate KYC’s pharmacodynamic profile but also provide biological confirmation of its system-level engagement in neonatal lung pathology.

#### 4.3.3. Systems Biological Effects of KYC in BPD

KYC has been shown to reversibly inhibit MPO activity [145]. It inhibits the peroxidase activity of MPO by entering the ferric protoporphyrin IX (heme) center to inhibit the binding of halides. The reaction between MPO and KYC transforms MPO into quasi-catalase and prevents HOCl formation. The tyrosine is an electron acceptor and becomes a tyrosyl radical after oxidation. The cysteine thiol group accepts the electron from the tyrosyl radical to become a thiyl radical. The thiyl radical reacts with thiol-containing molecules to form disulfide bonds. The quasi-catalase activity and inhibition of HOCl formation are not the only antioxidant properties of KYC. In the rat BPD model, we have shown that KYC also facilitates the expression of NRF2-induced antioxidant enzymes through thiylation and glutathionylation of KEAP1, followed by the stabilization of NRF2 [146]. The attenuation of ER stress is the third mechanism of the antioxidant activity [74]. These can explain why KYC decreases the chlorinated tyrosine and 8-hydroxy-deoxy-guanosine in BPD lungs.

The anti-inflammatory activities of KYC include decreasing neutrophil infiltration and MPO expression in the rat BPD lungs [146], reducing the expression of cyclooxygenases, TLR4, AGER, and extracellular HMGB1, oxidizing HMGB1, decreasing HMGB1 binding to PRRs [146], and attenuating ER stress [76], autophagy, and cellular senescence [31]. Fully oxidized HMGB1 loses its pro-inflammatory activity, and the decreased HMGB1 binding to TLR4, and AGER prevents the downstream NF-kB signaling. Attenuating autophagy, ER stress, and cellular senescence contribute to KYC’s anti-inflammatory properties.

In addition to its antioxidant and anti-inflammatory activities, KYC also increases the AT2 cell count in rat BPD lungs [31]. As AT2 behaves as a resident progenitor epithelial cell, the increased count by KYC indicates a better lung growth potential. Affymetrix studies show enrichment in the E2F, WNT/Catenin, and Notch signaling, with a decrease in TNFα/NF-kB, IL6/JAK/STAT3, P53/apoptosis, and epithelial–mesenchymal transition signaling that are awaiting further investigations. Mouse sickle cell models further demonstrate that KYC improves endothelial cell-mediated vasodilation, indicating an improved eNOS coupling [147]. The evidence suggests that KYC inhibits MPO, protects neonatal lungs against OS and inflammation, and offers more extensive benefits than vitamin A and caffeine. Thus, KYC operates not only as an anti-inflammatory and antioxidant agent but as a prototype for a next-generation class of therapeutics designed to operate within and reprogram the network dynamics of disease. Taken together, these findings not only validate KYC’s pharmacodynamic profile but also provide biological confirmation of its system-level engagement in neonatal lung pathology.

#### 4.3.4. Aligning KYC with the SPT Framework

Based on the characteristics and experimental findings discussed above, KYC satisfies multiple criteria that define a well-characterized SPT (Table 1). This is exemplified in the Table below as we map SPT attributes (as defined in Section 4.2) against KYC’s mechanisms and preclinical findings:

The development and characterization of KYC as an SCPD provides a tangible example of how SPT principles can be applied in neonatal disease contexts such as BPD. Its capacity to interface with the biochemical architecture of inflamed tissues and to exert controlled, multi-layered network effects points toward a new therapeutic horizon—one where complexity is not bypassed, but instead embraced, in drug design. As a first-in-class SCPD, KYC represents not just a therapeutic candidate for BPD but a model for rationally engineered network-aware drugs designed to restore homeostasis in complex disease systems.

## 5. Discussion

The dramatic advancement in neonatal care has successfully improved the survival rate of extremely premature neonates and contributed to the unchanged prevalence of BPD. Clinicians are facing an increasing number of survivors of premature neonates born at the late canalicular stage (22–26 weeks) of lung development [6,7]. Unlike diseases affecting other age groups, premature organs are still developing and require maintenance of their growth potential. The most significant contributors to BPD are OS, inflammation, and the use of a positive-pressure mechanical ventilator. Other contributors include fluid overload, patent ductus arteriosus, malnutrition, genetic factors, intrauterine growth restriction, and maternal smoking, among others. [148]. Although animal studies have helped us understand the mechanisms involved in the development and progression of BPD, it is not possible to study all risk factors, such as fluid overload, patent ductus arteriosus, and prolonged mechanical ventilation, in humans. Fortunately, all risk factors are associated with OS and inflammation.

Rodents are commonly used as small animal models for BPD [149,150]. Although rodent pups are born at the saccular stage, they are imperfect as they have no surfactant deficiency with mature antioxidant capacity. However, their large litter size, small body size, and relatively short life span make them the most convenient animal models. Researchers use LPS or hyperoxia (greater than 70% O_2_) to simulate inflammation and OS, respectively, to generate the BPD phenotype. We used outbred rat pups to study BPD, assuming they can better mirror the genetic diversity in the human population. Combining data from our experiments and those in the literature, we have constructed a theoretical “destructive cycle for BPD.” (Figure 1), which exhibits a self-perpetuating nature. The complexity of this cycle suggests that any therapy that only targets one mechanism will be insufficient in BPD treatment.

Available evidence suggests that caffeine and vitamin A have antioxidant and anti-inflammatory properties. The clinical success of early postnatal caffeine and high-dose vitamin A treatment in decreasing BPD incidence supports the importance of a multi-prong approach. Recent insights from systems pharmacology have highlighted the limitations of single-target drugs in addressing complex, nonlinear diseases, such as BPD. The concept of SPTs, which leverages network-aware, multinodal engagement, offers a promising strategy to overcome these limitations. In this context, our lead candidate, KYC, a first-in-class SCPD, represents a new therapeutic class capable of reprogramming disease networks. The deliberate mapping of KYC’s mechanism onto features of SPTs that includes modular engagement, context-specific activation, and emergent system-level effects supports its potential as a paradigm-shifting intervention in BPD.

## 6. Conclusions

BPD is a complex, multifactorial, and incompletely understood complication of RDS. As prematurity is not physiologic, decreasing OS and inflammation and preserving growth potential make BPD treatment different from the disease in other age groups. We propose the concept of utilizing SPTs to treat premature birth-associated disorders. In conclusion, integrating a systems pharmacology framework into the therapeutic landscape of BPD enables a more comprehensive and mechanistically coherent approach to treatment. Our work introduces KYC as a prototype SCPD, a molecule that conforms to the principles of an SPT by engaging disease-relevant networks in a spatially and biochemically restricted manner. The convergence of experimental evidence, mechanistic rationale, and systems-level design reinforces the promise of KYC not merely as a drug candidate but as a conceptual advance in how we approach pharmacotherapy for complex neonatal diseases.

## 7. Patents

1. SYSTEMS CHEMICO–PHARMACOLOGY DRUGS AND METHODS OF USE. Kirkwood Arthur Pritchard, Jr.; Dustin Paul Martin; Ru-Jeng Teng; Billy W. Day; and Stephen Naylor. Pub. No.: US 2022/0409692 A1 (NON-PROVISIONAL FULL APPLICATION).
2. DUAL ROLE COMPOUNDS WITH PRODRUG AND SYSTEMS CHEMICO-PHARMACOLOGY DRUG PROPERTIES. Billy W. Day; Kirkwood Arthur Pritchard, Jr.; Ru-Jeng Teng; and Stephen Naylor. FILED 04/21/2025 US PROVISIONAL PATENT APPLICATION 63/791,992.
3. SYSTEMS CHEMICO-BIOPROBES DERIVED FROM SYSTEMS CHEMICO-PHARMACOLOGY DRUGS FOR TARGET DISCOVERY AND COMPANION DIAGNOSTIC APPLICATIONS. Kirkwood Arthur Pritchard, Jr.; Billy W. Day; Ru-Jeng Teng; and Stephen Naylor. FILED 04/21/2025 US PROVISIONAL PATENT APPLICATION 63/792,015.

## Figures and Tables

**Table 1:** Aligning KYC with the SPT framework.

SPT Attribute	KYC Alignment
Multinodal Network Engagement	Modifies multiple proximal targets (e.g., HMGB1, Keap1) through a shared chemical mechanism (thiyl radical formation).
Context-Dependent Activation	Selectively activated by MPO in inflamed tissues, limiting off-target effects and enhancing safety.
Systemic Rebalancing	Reprograms redox and inflammatory signaling pathways (e.g., NRF2 activation, DAMP suppression).
Emergent Properties	Results in improved alveolarization and lung function not attributable to single-target inhibition.
Mechanism-Guided Design	Engineered with chemical features (thiol, tyrosine) for specific redox activation and network modulation.
Translational Scalability	Mechanistic pathway—MPO activation and downstream NRF2/DAMP effects—relevant to multiple diseases beyond BPD.

## Data Availability

The original contributions presented in the study are included in the article; further inquiries can be directed to the corresponding author.

## Abbreviations

The following abbreviations are used in this manuscript:

AGER	Advanced glycosylation end-product receptor
AT2	Type 2 alveolar epithelial cell
ATF6	Activating Transcription Factor 6
BiP	Binding immunoglobulin protein
BPD	Bronchopulmonary dysplasia
CD24	Cluster of differentiation 24
cGAMP	Cyclic guanosine monophosphate-adenosine monophosphate
CGAS	Cyclic GMP-AMP synthase
CHOP	C/EBP homologous protein
CXCR4	C-X-C motif chemokine receptor 4
DAMP	Damage-associated molecular pattern
eNOS	Endothelial nitric oxide synthase
ER	Endoplasmic reticulum
ETC	Electron transport chain
GCH1	GTP-cyclohydrolase 1
GRP78	Glucose-regulated protein 78
GSDMD	Gasdermin D
HAVCR2	Hepatitis A virus cellular receptor 2
HMGB1	High-mobility group box 1
HOCl	Hypochlorous acid
HSP	Heat-shock protein
iNOS	Inducible nitric oxide synthase
IRE1α	Inositol-requiring enzyme 1 α
JAK	Janus kinase
KEAP1	Kelch-like ECH-associated protein 1
KYC	N-acetyl-lysyltyrosylcysteine-amide
LPS	Lipopolysaccharide
MPO	Myeloperoxidase
mROS	ROS from mitochondria
MSC	Mesenchymal stem cell
NF-kB	Nuclear factor-kappa B
NLR	NOD-like receptors
NOX	NADPH oxidase
NRF2	Nuclear factor erythroid 2-related factor 2
ONOO^−^	Peroxynitrite
OS	Oxidative stress
PAMP	Pathogen-associated molecular patterns
PERK	PRKR-Like Endoplasmic Reticulum Kinase
PI3K	Phosphoinositide 3-Kinase
PPP	Pentose phosphate pathway
PRR	Pattern recognition receptor
PTEN	Phosphatase and tensin homolog
ROS	Reactive oxygen species
SAMD	Senescence-associated mitochondrial dysfunction
SASP	Senescence-associated secretory phenotype
SCPD	Systems chemico-pharmacology drug
SMAD	Suppressor of mothers against decapentaplegic.
SPT	Systems pharmacology therapeutics
STAT	Signal transducer and activator of transcription
STING1	Stimulator of interferon genes 1
TGFβ	Transforming growth factor β
TLR	Toll-like receptor
TNFα	Tumor necrosis factor α
TREM1	Triggering receptor expressed on myeloid cells 1
VEGF	Vascular endothelial cell growth factor
VEGFR2	Type 2 VEGF receptor
UPR	Unfolded protein response
WNT	Wingless/Integrated
XBP1	X-box binding protein 1
